# Dietary Acrylamide Exposure and Its Correlation with Nutrition and Exercise Behaviours Among Turkish Adolescents

**DOI:** 10.3390/nu17152534

**Published:** 2025-08-01

**Authors:** Mehtap Metin Karaaslan, Burhan Basaran

**Affiliations:** 1 Department of Nursing, Faculty of Health Sciences, Recep Tayyip Erdogan University, Rize 53100, Türkiye; 2 Department of Nutrition and Dietetics, Faculty of Health Sciences, Recep Tayyip Erdogan University, Rize 53100, Türkiye; burhan.basaran@erdogan.edu.tr

**Keywords:** adolescent, dietary acrylamide, nutritional behavior, exercise, non-carcinogenic risk, carcinogenic risk

## Abstract

**Background/Objectives:** Acrylamide is a probably carcinogenic to humans that naturally forms during the thermal processing of foods. An individual’s lifestyle—especially dietary habits and physical activity—may influence the severity of acrylamide’s adverse health effects. This study aimed to examine the relationship between adolescents’ dietary and exercise behaviors and their dietary acrylamide exposure and associated health risks. **Methods:** This descriptive and cross-sectional study was conducted with 370 high school students in Türkiye. Data were collected using the Nutrition Exercise Behavior Scale (NEBS) and a retrospective 24-h dietary recall questionnaire. Acrylamide exposure was calculated based on food intake to estimate carcinogenic (CR) and non-corcinogenic (target hazard quotient: THQ) health risks and analyzed in relation to NEBS scores. **Results:** Findings indicated that while adolescents are beginning to adopt healthy eating and exercise habits, these behaviors are not yet consistent. Emotional eating and unhealthy food choices still occur. Higher acrylamide exposure and risk values were observed in boys and underweight individuals. This can be explained mainly by the fact that boys consume more of certain foods—especially bread, which contains relatively higher levels of acrylamide—than girls do, and that underweight individuals have lower body weights despite consuming similar amounts of food as other groups. Bread products emerged as the primary source of daily acrylamide intake. Positive correlations were found between NEBS total and subscale scores and acrylamide exposure and health risk values. **Conclusions:** The study demonstrates a significant association between adolescents’ health behaviors and acrylamide exposure. These results underscore potential public health concerns regarding acrylamide intake during adolescence and emphasize the need for targeted nutritional interventions to reduce risk and promote sustainable healthy behaviors.

## 1. Introduction

Adolescence is a critical period of growth marked by mental, physical, and social changes that have significant effects throughout an individual’s life [1]. During this period, young people develop life skills, acquire positive health behaviors, and gain increasing autonomy in managing their own health [2]. However, negative health habits acquired during adolescence -particularly insufficient physical activity and unhealthy eating- pave the way for the development of noncommunicable diseases such as obesity and cardiometabolic diseases in later life [1,2,3].

Health behaviors such as nutrition and exercise play a key role in maintaining and improving adolescent health [2]. Engaging in at least 60 min of moderate to vigorous aerobic physical activity every day of the week provides numerous benefits for children and adolescents in terms of cardiovascular and muscular fitness, bone development, cognitive function, and mental health [4,5]. However, it is estimated that only 1 in 5 adolescents worldwide meet the WHO guidelines for physical activity [6].

On the other hand, it has been reported that diet quality tends to decline during adolescence [7,8]. Food preferences change over time based on biological, social, and environmental factors, and these preferences are among the key determinants of diet quality [9]. In a low-quality diet, consumption of ultra-processed foods such as high-sugar and high-fat foods, refined grains, processed meats, and ready-to-eat foods tends to increase, while consumption of healthy foods such as fruits, vegetables, whole grains, lean meats, and low-fat dairy products remains insufficient [10,11]. Indeed, the consumption of foods with low nutritional value is among the factors contributing to weight gain and obesity [1,3,12,13,14]. Additionally, a diet rich in ultra-processed foods increases the risk of exposure to various additives and harmful components formed during food processing [15]. One such risk is acrylamide, which naturally forms in foods during thermal processing [16,17].

Since its detection in certain foods by the Swedish National Food Administration in 2002, acrylamide has been considered a significant public health issue in the field of food safety [18]. Acrylamide forms at high levels during thermal processing at temperatures above 120 °C, particularly in carbohydrate-rich foods with low moisture content [19]. The fundamental mechanism behind this formation is the Maillard reaction between free asparagine and reducing sugars [20]. This compound can be found at varying levels in many functional and traditional foods commonly consumed in daily life, including potatoes and potato products, bread, coffee, and grain-based snacks [21,22,23,24,25,26]. However, the health effects of acrylamide ingested through food are much more important in terms of food safety [27]. The European Food Safety Authority (EFSA) has emphasized the genotoxic and carcinogenic potential of acrylamide, reporting that exposure levels are particularly high among children and individuals who consume ultra-processed foods [28,29]. EFSA and the Joint FAO/WHO Expert Committee on Food Additives (JECFA) have stated that acrylamide exposure through diet should be monitored and reduced to the lowest possible level [30,31]. Although there are studies in the literature that address adolescents’ dietary and exercise habits from different perspectives [32,33,34], to our knowledge, this study is the first to directly examine the relationship between dietary acrylamide levels and associated health risks and dietary and exercise behaviors in adolescents. The importance of this study is heightened by the fact that children and adolescents are more exposed to acrylamide through their diet than adults. The aim of this study is to (i) identify adolescents’ dietary and exercise behaviors, (ii) determine adolescents’ dietary acrylamide exposure and associated health risks, and (iii) examine and evaluate the relationship between adolescents’ dietary and exercise behaviors and acrylamide exposure and associated health risks.

## 2. Materials and Methods

### 2.1. Study Design and Population

This study was conducted using a descriptive and cross-sectional design in official public secondary education institutions located in the provincial center of Rize (Türkiye). Rize is a province in the northeast of Türkiye, on the Black Sea coast, and is one of the important centers of the Eastern Black Sea Region. The names of public schools located in the provincial center were listed, and five schools were randomly selected by lottery. Student lists were created at the class level in the selected schools, and simple random sampling was applied so that each student would have an equal chance of being selected. The number of students to be included from each school was determined in proportion to the total number of students in the school. The required sample size was determined using the G-Power 3.1.9.7 (Franz Faul, Universität Kiel, Kiel, Germany) analysis program; based on the results of the ANOVA test conducted using 99% power, 5% margin of error, and medium (0.25) effect size, it was deemed appropriate to include at least 345 participants.

The basic criteria for participation in the study were that participants had no known health problems, were able to communicate, and were willing to participate voluntarily. A total of 370 students who agreed to participate in the study by bringing a signed consent form from their parents were given the questionnaire, and all of them completed it. Due to the decrease in parental supervision, peer influence, and increased consumption of ready-to-eat foods outside the home during early and middle adolescence [35], as well as the fact that 11th and 12th graders in Türkiye are in an intensive exam preparation period, the surveys were administered to 9th and 10th graders, who were deemed more appropriate.

### 2.2. Data Collection Tools and Data Collection

The personal information form consists of questions about students’ demographic characteristics such as age and gender. Each student’s height and weight were measured and recorded on the form, and body mass index (BMI) was calculated by the authors using the formula: weight (kg)/height^2^ (m^2^).

In this study, height and weight measurements of students aged 13–16 were taken in the morning (08:30–10:00) in a school environment under standard and ethical conditions. Participants removed their shoes, coats, and heavy items and were measured wearing only their school uniforms and barefoot. Body weight was measured using a digital scale (Seca 813) placed on a flat surface and zeroed, and recorded with a precision of 0.1 kg when a stable value was observed. For height measurement, a non-stretching measuring tape fixed to the wall at ground level was used; students stood upright with the ruler placed at the top of their heads, and measurements were taken with a precision of 0.1 cm.

All anthropometric measurements in the study were performed by a single researcher. To ensure internal consistency of the measurements, each measurement was taken twice, and if there was a difference between the values, a third measurement was taken, and the mean of the two closest values was used. This approach was chosen to minimize measurement errors and increase the reliability of the data. The measurements were conducted in accordance with the World Health Organization (WHO) protocol [36].

The BMI assessments in adolescents were performed using BMI-age z-scores developed by the World Health Organization. These z-scores were calculated using the WHO’s free AnthroPlus 1.0.4 software, based on the students’ age (in months) and gender. A z-score of <−2 is classified as underweight, −2 to +1 as normal, +1 to +2 as overweight, and z ≥ +2 as obese. This method allows for a more sensitive, meaningful, and internationally comparable analysis of nutritional status, taking into account the rapid growth and developmental differences during adolescence [37].

In this study, the Nutrition-Exercise Behavior Scale (NEBS) was used as a behavioral indicator of dietary acrylamide exposure. Because the NEBS assesses behaviors such as healthy dietary choices, meal planning, and avoiding processed foods, it indirectly reflects dietary trends that may be associated with acrylamide exposure. The NEBS was developed by Yurt et al. [38]. The scale has 45 questions and 4 sub-dimensions. Scale scores are evaluated based on the scores obtained from the scale sub-dimensions. The score range for the psychological/addictive eating behavior (PAEB) subfactor is 11–55, with a high score indicating psychological/addictive eating behavior. The score range for the healthy eating-exercise behavior (HEEB) subfactor is 14–70, with a high score indicating healthy eating-exercise behavior. The score range for the unhealthy eating-exercise behavior (UEEB) subfactor is 14–70, with a high score indicating unhealthy eating-exercise behavior. The score range for the meal regularity (MR) subfactor is 6–30, with a high score indicating good meal regularity.

The overall original Cronbach’s alpha (α) reliability coefficient of the NEBS scale was reported as 0.85, while the subscale coefficients were reported as 0.61, 0.62, 0.68, and 0.73, respectively. In the current study, the overall Cronbach’s alpha (α) reliability coefficient of the scale was determined to be 0.81, while the subscale reliability coefficients were 0.82, 0.78, 0.72, and 0.74, respectively. 0.00 < α < 0.40 scale is not reliable, 0.40 < α < 0.60 low reliability, 0.60 < α < 0.80 considerable reliability, 0.80 < α < 1.00 high reliability [39]. Therefore, it can be said that the scale is quite reliable and highly reliable. Furthermore, since the original language of the scale is Turkish, no translation or cultural adaptation was necessary.

### 2.3. Dietary Questionnaires

In this study, the 24-h recall method was preferred to facilitate adolescents’ recall of their consumption patterns of bread, desserts, tea, and coffee and to obtain accurate consumption data. In the first section of the questionnaire, the purpose of the study was defined, and descriptive questions were asked to determine the adolescents’ gender, age, body weight, and height. In the second section, adolescents were asked whether they had consumed bread, desserts, tea, and coffee from the time they woke up in the morning until they went to bed at night, and the consumption amounts of participants who indicated that they had consumed these foods were recorded. The portion sizes of the foods consumed were determined using a food and nutrition photo catalog. It took approximately 40–45 min for participants to record all the data.

### 2.4. Acrylamide Levels in Food

This study included three food groups. The selection of bread, desserts, and beverages as the target food groups in this study was based on three main criteria: (1) their high and frequent consumption in the adolescents, (2) their substantial contribution to total dietary acrylamide intake in prior dietary surveys, and (3) their representation of diverse food matrices (solid, semi-solid, and liquid), which allowed for a more comprehensive exposure model. Although it is well documented that fried snacks and processed cereals can contain high acrylamide levels, these foods were excluded because standardized and up-to-date acrylamide concentration values for these products were not available at the time of the study. The acrylamide concentrations used in this study were obtained from previous studies conducted in Türkiye and reflect widely consumed local products such as white bread, traditional desserts, and nationally available brands of tea and coffee (Table 1).

### 2.5. Dietary Acrylamide Exposure

In this study, the level of acrylamide exposure resulting from adolescents’ consumption of bread, desserts, and beverages was calculated using the following Equation (1) [43].

(1) $$\mathrm{EDI}=\frac{{\;H}_{x}\;\times {\;H}_{y}\;}{{\;b}_{w}\;\times \;\mathrm{CF}}$$

EDI is the estimated daily acrylamide exposure (µg/kg/day), H_x_ is the daily intake for each food (mL-g/day), H_y_ is acrylamide concentration of each food (µg/mL-kg), CF (Conversion factor) = 1000, and b_w_ is the body weight (kg; the body weight of each adolescent included in the study was used).

### 2.6. Carcinogenic and Non-Carcinogenic Health Risk Assessment

HI (Hazard index) describes the non-carcinogenic health risk. HI is the sum of the THQ (Target Hazard Quotient) values calculated for the acrylamide exposure level resulting from the consumption of each food. HI and THQ have no units. THQ or HI value ≥ 1 indicates a potential non-carcinogenic health risk, while a value <1 implies that the associated health hazard is negligible [44]. THQ and HI were calculated using the following Equations (2) and (3), respectively.

(2) $$\mathrm{THQ}=\frac{\mathrm{EDI}}{\mathrm{RfD}}$$

(3) $$\mathrm{HI}=\sum {THQ}_{Bread}+{THQ}_{Dessert}+{THQ}_{Beverage}$$

EDI is the estimated daily acrylamide exposure (µg/kg/day), RfD is oral reference dose. The United States Environmental Protection Agency defined the RfD determined for acrylamide related to degenerative nerve changes is 2 µg/kg/day [45].

Carcinogenic risk (CR) was determined using the following Equation (4).

(4) CR=EDI × OSF

EDI is the estimated daily acrylamide exposure (µg/kg/day), oral slope factor (OSF) determined for acrylamide is 500 µg/kg/day [45]. According to the United States Environmental Protection Agency, a CR value below 1.00 × 10^−6^ is considered safe; a value between 1.00 × 10^−6^ and 1.00 × 10^−4^ indicates a potential risk; and a value exceeding 1.00 × 10^−4^ represents a serious health risk [46].

### 2.7. Statistical Analysis

The study data were transferred to IBM SPSS Statistics 26.0 (IBM, Armonk, NY, USA) for analysis. When evaluating the data, frequency distributions were provided for categorical variables, and descriptive statistics (mean, standard deviation, median) were provided for numerical variables. The reliability of the NEBS, which was used as a measurement tool in the study, was examined using Cronbach’s alpha internal consistency coefficient.

The total NEBS scores and subscale scores of the individuals participating in the study were obtained by summing the relevant items. Accordingly, in order to decide on the analyses to be applied, the Kolmogorov-Smirnov test (*n* > 30) was first applied to all scores and exposure measurements to test the assumption of normal distribution. The test results showed that the NEBS total score and subscale scores met the assumption of normal distribution, while the exposure measurements did not. Therefore, both parametric and nonparametric tests were used in the comparisons.

The Independent Samples *t-* Test and Mann Whitney U Test were used to examine whether there were differences between two independent groups based on scores and measurements. The One-Way Analysis of Variance (ANOVA) and Kruskal Wallis Test were used to examine whether there were differences between more than two independent groups based on scores and measurements, and the Tukey Test and Bonferroni Test were used to determine which groups differed from each other. The Spearman’s rho Correlation Coefficient was used to determine the degree of non-causal relationships between two numerical variables. In correlation coefficient (r) calculations, the coefficients range from −1 to 1. r = 0.00 (no correlation), r = 0.01–0.29 (low correlation), r = 0.30–0.69 (medium correlation), r = 0.70–0.99 (high correlation), r = 1.00 (full correlation). Different letters within the same group indicate statistically significant differences (*p* < 0.05) [47].

## 3. Results

### 3.1. Demographic Characteristics of Adolescents

A total of 370 adolescents were included in the study. The adolescents were aged between 13 and 16 years. The majority of adolescents were of normal weight. The mean age, height, body weight (girls 54.09 ± 9.72 kg; boys 63.73 ± 12.50 kg), and BMI values were 14.1 ± 0.49, 165 ± 7. 73 cm, 58.6 ± 12.1 kg, and 21.4 ± 3.53 kg/m^2^, respectively (Table 2).

While boys consumed more bread and desserts than girls (*p* < 0.05), no statistically significant difference was found between the two groups in terms of beverage consumption (*p* > 0.05). According to BMI, the bread consumption levels of adolescents in the overweight/obese and underweight groups were higher than those in the normal weight group, while the dessert and beverage consumption levels of adolescents in the overweight/obese group were higher than those in the normal and underweight groups (*p* < 0.05).

### 3.2. NEBS and Sub-Dimensions in Adolescents

The mean NEBS score of the individuals participating in the study was 133 ± 20.3 (77–202) The mean PAEB subscale score was 29.3 ± 9.33 (11–55), the mean HEEB subscale score was 43.3 ± 9.78 (18–69), the mean UEEB subscale score was 38.9 ± 7.63 (19–62), and the mean MR subscale score was 21.4 ± 5.19 (6–30) (Table 3). No statistically significant difference was found between BMI groups based on NEBS total scores and subscales (*p* > 0.05). The mean NEBS total scores, HEEB scores, and MR scores of girls were significantly lower than those of boys (*p* < 0.05) (Table 3).

### 3.3. Dietary Acrylamide Exposure and Health Risk Assessment

The results of the analysis examining whether the acrylamide exposure level, THQ and CR values of adolescents resulting from the consumption of bread, desserts and beverages differed in terms of demographic characteristics are shown in Table 4.

The level of acrylamide exposure in boys due to their consumption of bread, desserts, and beverages, and the associated carcinogenic and non-carcinogenic health risk values are higher than in girls. By gender, the daily/body weight/total acrylamide exposure level, THQ, HI, CR, and total CR values resulting from bread and dessert consumption are higher in boys than in girls (median; *p* < 0.05). There was no statistically significant difference between genders in terms of acrylamide exposure levels, THQ, and CR values from beverage consumption (median; *p* > 0.05).

According to BMI, acrylamide exposure, THQ, and CR values based on body weight due to beverage consumption are higher in adolescents in the underweight group than in those in the overweight/obese group (median; *p* < 0.05). Total acrylamide exposure, HI, and CR values based on body weight are higher in individuals in the underweight group than in adolescents in the normal weight and overweight/obese groups (median; *p* < 0.05).

**Figure 1:**
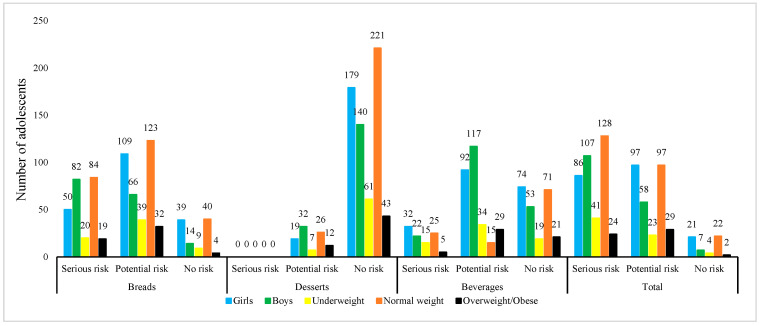
Individual risk categories of adolescents based on CR values. A z-score of <–2 is classified as underweight, –2 to +1 as normal, +1 to +2 as overweight, and z ≥ +2 as obese [37].

Data on risk categories for adolescents according to CR values are shown in Figure 1.

The results of the correlational analysis between the scores obtained for NEBS and its sub-dimensions and the acrylamide exposure based on diet and related health risk values are shown in Table 5.

The results reveal significant correlations between daily acrylamide exposure, acrylamide exposure based on body weight, THQ, and CR levels with NEBS, PAEB, and UEEB. 

## 4. Discussion

### 4.1. Adolescents’ Nutrition and Exercise Behaviors

The scores obtained in this study regarding adolescents’ nutrition and exercise behaviors indicate that healthy habits have generally been adopted, but these behaviors have not yet been fully established. This can be explained by the mean PAEB, HEEB, UEEB, and MR subscale scores calculated for adolescents. As the subscale scores increase, adolescents exhibit more positive and negative behaviors. The mean values of the negative behaviors PAEB and UEEB (29.3 and 38.9, respectively) were found to be lower than the median value (33 and 43, respectively) when considering the score ranges determined for these subscales (11–55 and 14–70, respectively). On the other hand, the mean values of the positive behaviors HEEB and MR (43.3 and 21, respectively) were found to be relatively higher than the median value (43 and 18, respectively) when considering the score ranges determined for these subscales (14–70 and 6–30, respectively). The findings reveal the existence of psychological eating tendencies and occasional unhealthy preferences, which is consistent with other studies in the literature. Akdeniz Kudubes et al. noted that adolescents’ nutrition and exercise behaviors exhibit limited positive characteristics. Similarly [34], Ayaz-Alkaya and Kulakçı-Altıntaş emphasized that individuals in this age group are in the process of developing healthy behaviors [2].

In a study conducted by Sarı and Ceylan, it was stated that adolescents’ tendencies toward nutrition and exercise behaviors are in the developmental stage [33]. Although adolescents generally exhibit positive nutrition and exercise behaviors, the findings show that participants sometimes turn to psychological eating and unhealthy choices. Adolescence is a period of emotional fluctuations and risky behaviors [48]. Although individuals’ genetic structures cannot be changed, healthy lifestyle habits can be developed by making adjustments to eating behaviors, physical activity levels, and other environmental factors [34]. Emotional fluctuations can be reduced through regular physical activity [49,50,51], thereby reducing adolescents’ psychological eating preferences. Indeed, children who engage in planned and regular physical activity have been found to have higher Healthy Eating-Exercise Behaviors than those who do not engage in planned and regular physical activity [52]. Yarar et al. found that healthy eating and exercise behaviors were more prevalent among adolescent athletes than unhealthy eating and exercise behaviors [53]. These findings show that despite the promotion of healthy lifestyle behaviors, there is still room for improvement, particularly in areas such as emotional eating and unhealthy habits. Additionally, participants’ attention to meal schedules stands out as a positive finding. The analyses show that girls tend to have lower total nutrition-exercise behavior scores, lower healthy nutrition-exercise behavior, and more irregular meal consumption than boys. Aykut et al. also stated that girls have a more irregular meal schedule [54]. Weight satisfaction and weight perception play an important role in shaping eating behaviors [55]. Negative body image among adolescents can be an unhealthy factor influencing dietary decisions [56], and its impact is increasing, especially among girls [57]. Girls may engage in unhealthy behaviors such as skipping meals and following strict diets in order to control their weight due to body image concerns. However, it should be noted that girls lower scores may be due not only to a lack of motivation but also to structural barriers such as concerns about suitable venues and safety for physical activity, increased responsibilities related to social roles, or time constraints. On the other hand, boys adoption of a regular and active lifestyle [58] may facilitate their having “higher nutrition-exercise behavior” scores. The findings indicate that cultural and environmental factors, body image, and psychological factors must also be taken into account in order to thoroughly examine gender-based differences. More comprehensive studies with qualitative data could shed more light on the underlying reasons for differences in nutrition and exercise behaviors between girls and boys. This would enable the development of targeted intervention programs that take into account the needs and barriers of each gender.

The current study found no significant effect of BMI on nutrition and exercise behaviors, which is consistent with study in the literature that have found no relationship between BMI and nutrition and exercise habits [54]. On the other hand, other studies have demonstrated that an increase in BMI is associated with an increase in psychological dependence on eating behavior and a decrease in healthy nutrition and exercise behaviors, thereby arguing that there is a strong relationship between BMI and nutrition and exercise behaviors [33,59]. These differences may stem from the sociodemographic characteristics of the samples, the measurement tools used in the studies, and the variety of data collection methods. In addition, factors such as cultural factors, individual motivation levels, and health literacy may also play a decisive role in the relationship between BMI and nutrition-exercise behaviors. Therefore, to clarify the reasons behind conflicting findings and achieve clearer results, more comprehensive and multidimensional studies conducted on different samples are necessary.

### 4.2. Dietary Acrylamide Exposure

In this study, dietary acrylamide exposure from three major food groups—bread, desserts, and beverages—was evaluated according to demographic variables, including gender and BMI.

When evaluated by gender, it was observed that boys individuals had higher exposure values than girls individuals in all three food groups. In particular, the mean daily acrylamde exposure of boys in the bread group was 13.8 ± 11.9 µg/day, while the value for girls was 5.65 ± 7.02 µg/day. Similarly, a similar trend is observed in acrylamide exposure values based on body weight (0.23 ± 0.21 µg/kg/day for boys; 0.11 ± 0.14 µg/kg/day for girls) (Table 4). This difference can be attributed to boys having higher energy levels and therefore consuming more bread. Indeed, the boys included in this study consumed on mean more than twice as much bread as the girls (Table 2). The higher level of acrylamide exposure in boys compared to girls due to bread consumption is consistent with the literature. Mojska et al. reported the mean acrylamide exposure from bread consumption for children and adolescents aged 7–18 years as 12 and 9.3 µg/kg/day for boys and girls, respectively [60], Acrylamide exposure from bread consumption in Türkiye has been reported to be 0.34 and 0.20 µg/kg/day for boys and girls aged 15–18, respectively [40].

The mean daily acrylamide exposure levels from dessert consumption for boy and girls were calculated as 0.51 ± 1.23 µg/day (0.00 ± 0.02 µg/kg/day) and 0.21 ± 0.77 µg/day (0.01 ± 0.02 µg/kg/day), respectively. In both gender groups, acrylamide exposure from dessert consumption is generally lower than that from other food groups (Table 4). The main reason for this situation is related to the low place and importance of desserts in daily nutrition. The main reason for the statistically significant difference between genders is related to boys consuming more desserts than girls (Table 2). This situation is similarly related to boys having higher daily energy requirements. In France, the mean acrylamide exposure from the consumption of cakes and other sweetened pastries among children and adolescents aged 3–17 is 0.026 µg/kg/day [61], while in Poland, the mean acrylamide exposure level from cheesecake consumption among individuals aged 18–36 is reported to be 0.030 µg/kg/day [62]. In this study, the level of acrylamide exposure from dessert consumption among adolescents is lower than in other studies. The main reason for this is thought to be related to the fact that the acrylamide content of reported desserts varies and that dessert consumption varies according to geography and culture.

No significant gender-related differences were found in the data related to the beverage group. Both daily acrylamide exposure values (mean = 5.36 ± 5.59 and 5.69 ± 5.21 µg/day, respectively) and acrylamide exposure values based on body weight (mean = 0.09 ± 0.09 and 0.10 ± 0.11 µg/kg/day, respectively) were quite similar in boys and girls (Table 4). This situation can be explained by the fact that beverage consumption levels are similar in both genders (Table 2). While the daily acrylamide exposure level from beverage consumption is higher in boys, the acrylamide exposure level relative to body weight is higher in girls. The main reason for this is that girls have a lower body weight than boys. Zha et al. and Kito et al. found similar values for acrylamide exposure from tea consumption, at 0.03 µg/kg/day [63,64]. In another study, acrylamide exposure from tea consumption among males and females aged 15–18 years was reported to be 0.13 and 0.10 µg/kg/day, respectively [65] Acrylamide exposure from coffee consumption in children and adolescents has been reported as 0.007 µg/kg/day in Poland [60] and 0.001–0.002 µg/kg/day in France [61]. In this study, since acrylamide exposure from beverage consumption was calculated together for tea and coffee, the findings can be said to be consistent with the literature.

According to the BMI values of adolescents, the daily acrylamide exposure level from bread consumption was determined to be the lowest and highest in the normal and overweight/obese groups, respectively. In contrast, the acrylamide exposure level based on body weight was highest in underweight individuals at 0.22 ± 0.23 μg/kg/day, while this value was 0.15 ± 0.17 and 0.15 ± 0.14 μg/kg/day in normal and overweight/obese individuals, respectively. Acrylamide exposure levels from dessert consumption were lower than those from other food groups in all BMI categories. Differences in acrylamide exposure levels from beverage consumption between BMI groups were limited. The level of acrylamide exposure based on body weight was found to be highest in the underweight group in both the dessert and beverage groups (Table 4). According to the total daily acrylamide exposure level, the BMI categories are ranked as overweight/obese > underweight > normal weight. However, it was observed that underweight individuals were exposed to acrylamide at higher levels relative to their body weight. This finding shows that when consuming similar amounts of food, underweight individuals are exposed to more acrylamide per unit weight due to their smaller body mass. No study examining acrylamide levels in adolescents based on BMI values has been found in the literature. However, it is known that individuals with low body weight are more exposed to diet-related toxic compounds such as acrylamide.

When the total acrylamide exposure of adolescents was evaluated according to gender and BMI values, the total daily acrylamide exposure values for boy and girls were 20.0 ± 14. 1 and 11.2 ± 9.49 µg/day, respectively, while their total acrylamide exposure levels based on total body weight were calculated as 0.33 ± 0.25 and 0.21 ± 0.19 µg/kg/day, respectively. The acrylamide exposure levels based on total body weight for adolescents in the low, normal, and overweight/obese groups were calculated as 0.35 ± 0.29, 0.25 ± 0.21, and 0.23 ± 0.19 μg/kg/day, respectively. In this study, the acrylamide levels reached for adolescents in both gender and BMI categories were lower than the tolerable daily intake levels for acrylamide neurotoxicity and cancer (40 and 2.6 μg/kg/day, respectively) [66] and lower than the values defined by JECFA for the general population and consumers with high exposure (1 and 4 µg/kg bw/day, respectively) [30].

The contribution levels of food groups to the total daily acrylamide exposure of boys were 65.4, 6.1, and 28.5% for bread, desserts, and beverages, respectively, while the contribution levels for girls were 50.4, 1.6, and 48%, respectively. According to BMI categories, the contribution levels of bread, desserts, and beverages to total daily acrylamide exposure are 64.3, 2.3, and 33.4% for the underweight group, 60.1, 1.9, and 38% for the normal weight group, and for the overweight/obese group, they are 64.7%, 4.1%, and 31.2%, respectively. These ratios reveal that bread consumption contributes most significantly to acrylamide exposure in adolescents. Indeed, numerous studies in the literature have reported that bread consumption contributes 25–40% to daily acrylamide exposure [60,67,68]. Bread is one of the staple foods in many different countries and regions. The Türkiye Nutrition and Health Survey reported that the mean bread consumption in Türkiye is 180 g [69]. It has been reported that Türkiye is one of the countries with the highest bread consumption in the world [70]. This information explains the high contribution level of acrylamide exposure from bread consumption.

The beverage group includes tea, Turkish coffee, instant coffees, and ready-to-drink coffees. Tea is thought to contribute significantly to acrylamide exposure levels. This is because Türkiye ranks first in the world in terms of per capita tea consumption, at 4 kg [40]. In addition to Turkish coffee being a traditional beverage, instant coffees being easy to prepare, and these products being readily available in many markets, including school cafeterias, the consumption of ready-to-drink coffees has also become widespread in recent years with the increasing popularity of modern coffee shops. Therefore, the second highest contribution to daily acrylamide exposure comes from beverages. In the literature, the contribution of beverage consumption, particularly coffee, to daily acrylamide exposure has been reported at high rates of 15% [71], 27% [72], and 40% [73]. However, there are also studies reporting that coffee consumption contributes to the acrylamide exposure level of individuals aged 7–18 based on nutrition at a very low level of 1% [60]. However, it is thought that age is a factor influencing tea and coffee consumption habits.

In Türkiye, desserts are mostly consumed on special occasions or with meals. It can be said that the limited role of desserts in daily nutrition affects the level of contribution to acrylamide exposure levels. Many studies have also reported that dessertconsumption contributes to daily acrylamide exposure at a low level [61,68]. Therefore, the findings for desserts are consistent with the literature.

Effective strategies to reduce acrylamide formation have emerged at both industrial and household levels. Industrial methods include temperature optimization, shorter cooking times, and asparaginase use, while household practices such as boiling instead of frying or roasting can lower exposure. Applying these measures in daily routines helps translate research into practical dietary and public health actions.

### 4.3. Carcinogenic and Non-Carcinogenic Health Risk Assessment

When mean THQ values are examined according to gender and BMI categories, it is seen that boys have higher values than girls, and adolescents in the underweight group have higher values than other groups (Table 4). However, it was determined that the mean, minimum, and maximum THQ values calculated for each food in all groups, as well as the total THQ (HI) values, were significantly lower than the critical limit of 1. Therefore, the findings indicate that the level of acrylamide exposure from bread, dessert, and beverage consumption is safe in terms of non-carcinogenic health risks. However, the fact that the HI value calculated for maximum THQ values can be up to 0.67 in some individuals indicates that particular attention should be paid to high consumption scenarios. Studies conducted on adults in Iran and young people in Türkiye have determined THQ values for acrylamide exposure from bread consumption to be 0.06 and 0.10/0.17 (female/male), respectively [40,74]. The mean THQ values for tea consumption were 0.07 and 0.05 for males and females aged 15–18, respectively [65], while the mean THQ value for coffee consumption was reported to be 0.9–0.24 [75]. In another recent study, two different scenarios were developed, one good and one bad, and the THQ values for bread, tea, coffee, and dessert consumption were determined to be 0.11/0.11, 0.12/0.12, 0.003/0.003, and 0.09/0.01, respectively [68]. Therefore, the findings obtained for THQ and HI in this study can be said to be generally consistent with the literature.

When examining the mean CR values for each food group and the total CR values, it is noteworthy that the means are higher for boys than for girls. The mean CR values for boys indicate that bread consumption poses a serious health risk in terms of carcinogenicity, while consumption of desserts and beverages poses potential health risks. For girls, the mean CR values indicate that bread, dessert, and beverage consumption pose potential health risks. Based on the mean total CR values, boys face a serious health risk from a carcinogenic perspective, while girls face a potential health risk. In a study conducted in Türkiye, the mean CR value (1.0 × 10^−4^–1.7 × 10^−4^) resulting from bread consumption among individuals aged 15–18 was reported to be higher than in this study [40]. In two separate studies conducted in Iran, CR values were reported as 1.83 × 10^−4^–11.1 × 10^−4^ [72] and 7.6 × 10^−4^ [76]. Basaran calculated CR values for acrylamide exposure from bread, tea, coffee, and dessert consumption in adults, with values of 1.05 × 10^−4^/1.05 × 10^−4^, 1.19 × 10^−4^/1.19 × 10^−4^, 3.45 × 10^−6^/3.45 × 10^−6^, and 1.23 × 10^−5^/8.64 × 10^−5^, respectively [68]. The CR value calculated for tea consumption in this study was considered significantly higher compared to that study. However, it is thought that the main reason for the difference between the two studies is that the target group in this study was adolescents, whereas the target group in the relevant study was adults. When the mean CR values for each food group and the total CR values were examined according to BMI categories, it was observed that adolescents in the underweight group had higher CR values than those in other groups. The mean CR values for bread consumption indicate a serious carcinogenic risk for adolescents in the underweight group and a potential carcinogenic health risk for adolescents in the normal and overweight/obese groups. The mean CR values for dessert and beverage consumption indicate a potential carcinogenic health risk for all BMI categories.

When mean total CR values are considered, it is understood that adolescents in the low, normal and overweight/obese groups face serious risks, in terms of carcinogenicity. No study has been found in the literature that calculates the CR value for acrylamide exposure based on BMI values in adolescents.

Within the scope of the study, CR values were also evaluated by considering the possibility that adolescents may individually consume higher amounts of the target foods. Accordingly, 48% of boy and 25% of girls are exposed to acrylamide through bread, while 13% and 16% respectively are exposed through beverages—both posing serious carcinogenic health risks. Since none of the boy and girls had a CR value greater than 1 × 10^−4^ from dessert consumption, no serious carcinogenic health risk was identified. However, 19% of boys and 10% of girls have CR values indicating potential health risks due to their consumption of desserts. When CR values are evaluated in terms of daily total acrylamide exposure levels, 62% of boys and 43% of girls are at serious health risk in terms of carcinogenicity. When the total CR values of adolescents in the low, normal, and overweight/obese groups are considered, 60%, 52%, and 44% of them, respectively, carry serious health risks in terms of carcinogenicity, while 34%, 39%, and 53% of them, respectively, carry potential health risks. The proportion of adolescents who do not pose any carcinogenic health risk is quite low (Figure 1). All these findings indicate that measures should be taken to reduce acrylamide exposure in both boy and girls and that the risk is significant. THQ and CR values are directly related to the level of acrylamide exposure resulting from food consumption. Therefore, in order to reduce the health risks associated with acrylamide exposure, it is necessary to reduce food consumption or to include more foods with low acrylamide levels in the diet.

Although some THQ and CR values in this study fell below established risk thresholds, this does not rule out potential health concerns. Chronic low-level exposure—especially in vulnerable groups like adolescents—may still contribute to long-term risks. Thus, these findings should be viewed as precautionary signals rather than conclusive evidence of safety. Non-significant subgroup differences may reflect sample size limitations or exposure variability. In this context, THQ and CR serve as early warning indicators to guide risk prioritization and inform dietary strategies to reduce acrylamide exposure in adolescents.

### 4.4. Relationship Between Dietary Acrylamide Exposure, THQ and CR Values, and Nutritional and Exercise Behaviors and Their Sub-Dimensions

A low-level but significant positive linear correlation was found between total NEBS scores and acrylamide exposure from bread and desserts (both daily and body weight–adjusted), as well as THQ and CR values. A similar correlation was also observed between total acrylamide exposure (daily and per body weight), THQ, and CR values (Table 5). A low-level positive linear relationship was found between the total score for NEBS and daily acrylamide exposure from beverage consumption (Table 5). This indicates that as the dietary and exercise behavior score increases, even at a low level, individuals tend to consume more bread and desserts and therefore have higher acrylamide exposure. It is understood that the presence of bread at every meal and its high consumption in the Turkish diet contribute to this situation.

A low-level positive linear relationship was found between the PAEB score and the total daily acrylamide exposure level based on bread and dessert consumption, as well as body weight, THQ, and CR values (Table 5). However, correlation values for the beverage group were very low and not statistically significant (*p* > 0.05, Table 5). This suggests that, although the effect size is low, the relevant subscale has a greater influence on individuals’ specific food preferences and appears to be particularly associated with the consumption of products such as bread and desserts. Eating behavior is shaped not only in response to physiological needs but also in direct relation to the individual’s psychological state, stress level, and mood fluctuations [77]. In this context, psychological eating behavior—the individual’s need to eat outside of physical hunger, usually triggered by stress, anxiety, or emotional deprivation—has been intensively researched in the fields of nutrition and psychology in recent years [78,79,80]. This behavior is particularly characterized by a preference for carbohydrate-rich foods such as bread and desserts [81]. Refined carbohydrates activate the brain’s reward system by triggering dopamine release [82]. This biochemical cycle can lead individuals to develop tolerance to such foods and enter a cycle of repeated consumption [83]. Indeed, neuroimaging studies have shown that high-sugar and high-glycemic index foods activate similar brain regions as alcohol or drug use [84]. Additionally, carbohydrate consumption provides temporary emotional relief by increasing serotonin levels [85]. The positive relationship between high carbohydrate consumption and emotional eating has been supported by various studies [86]. In individuals exhibiting psychological addictive eating behavior, the consumption of carbohydrate-rich foods has been observed to increase independently of physical hunger, and this has been shown to lead to both psychological and metabolic issues over time [87]. Additionally, health risks associated with dietary acrylamide exposure may increase in individuals. Therefore, carbohydrate-based psychological eating behavior should be addressed not only as a nutritional issue but also as an area for psychological intervention due to potential health risks associated with acrylamide, and should be evaluated using multidisciplinary approaches.

A low but statistically significant positive correlation was found between UEEB scores and acrylamide exposure levels—both daily and body weight–adjusted—including total exposure from bread, desserts, beverages, and the associated health risks (Table 5). Although the strength of this relationship is weak, the findings suggest that this subscale may reflect a tendency toward the consumption of carbohydrate-rich and beverage-based processed foods, which are known sources of acrylamide. The growing prevalence of such dietary patterns contributes not only to nutritional imbalances but also to increased exposure to potentially carcinogenic and neurotoxic compounds, posing a significant public health concern. Therefore, it is expected that unhealthy eating habits and the consumption of foods high in acrylamide content are positively associated with acrylamide exposure and related health risks. Unhealthy eating habits are generally defined as the preference for energy-dense, nutrient-poor, fiber-deficient, and highly processed foods [88]. The most characteristic foods within this behavioral pattern are also rich in acrylamide. Indeed, the foods included in this study from the bread, dessert, and beverage groups confirm this information. Individuals with unhealthy eating behaviors are drawn to foods that are fast, tasty, and easily accessible [89,90]; however, it has been observed that many of these foods have high acrylamide content [91]. In addition, the replacement of home cooking habits with the consumption of prepared foods from outside sources can also be considered a factor that increases acrylamide exposure. At the societal level, the spread of unhealthy eating habits, especially among the younger population, should be evaluated not only in terms of the risk of obesity and metabolic syndrome, but also in terms of acrylamide exposure, which is a potential carcinogenic compound for humans. This situation points to the need to develop multi-layered strategies addressing the long-term risks of unhealthy eating.

No significant correlation was found between HEEB scores and dietary acrylamide exposure or related health risk indicators (Table 5), suggesting that this sub-dimension is not a decisive factor across food groups. While healthy eating behaviors are crucial for maintaining nutrient balance and overall health, they also play a role in minimizing exposure to harmful compounds. In this context, healthy eating may inversely relate to acrylamide exposure and its associated risks, although this was not supported statistically in the present study. Healthy eating behavior encompasses basic principles such as meeting an individual’s energy needs in a balanced manner, prioritizing fresh and unprocessed foods, preferring low-temperature cooking methods such as boiling and steaming, and avoiding processed convenience foods [92]. Therefore, healthy eating behavior directly excludes dietary patterns that include foods high in acrylamide content. This behavior model is also valuable in terms of preventing obesity, diabetes, and cardiovascular diseases [93,94]. Additionally, individuals who exhibit healthy eating behavior generally prioritize reading food labels, health literacy, and conscious consumption behaviors [95,96]. As a result, individuals tend to avoid not only acrylamide but also other similar harmful components due to their dietary preferences. From a societal perspective, promoting healthy eating habits can play a strategic role in preventing exposure to chemicals such as acrylamide. At this point, education policies and school nutrition programs should come into play.

The MR subscale exhibited low, non-significant correlations in both directions (Table 5), indicating that it is not a meaningful predictor of acrylamide exposure, related health risks, or dietary behavior patterns examined in this study. The quality of dietary behaviors is directly related not only to the content of the foods consumed but also to the time intervals and pattern in which these foods are consumed. Meal pattern is a fundamental dietary structure that enables individuals to meet their daily energy and nutrient requirements over time [97]. Disruption of this pattern has the potential to indirectly increase acrylamide exposure and related health risks by disrupting macronutrient balance and increasing the consumption of unhealthy and fast foods. Current research shows that individuals who skip meals or go without food for long periods of time experience blood sugar imbalances, weak appetite control, and sudden energy needs, which in turn increase their tendency to consume foods with a high glycemic index and industrially produced foods [98,99,100,101,102]. This behavioral pattern places foods with a high risk of acrylamide formation at the center of the diet. Individuals with a regular meal schedule, on the other hand, often make conscious food choices and exhibit healthy eating habits [103,104,105]. Therefore, meal patterns are an important determinant not only of energy balance but also of exposure to harmful compounds such as acrylamide. This situation threatens healthy living at the individual level and increases the burden on public health at the societal level. For this reason, nutrition education programs should not only cover what to eat, but also when and how to eat; maintaining meal patterns should be adopted as an important strategic goal in protecting against thermal processing contaminants such as acrylamide that threaten human health.

An individual’s dietary habits are the primary determinant of acrylamide exposure. However, physiological factors other than diet also affect the body’s ability to cope with this toxic substance. While a direct causal link between physical activity and acrylamide intake is not yet established, exercise can influence both food choices and metabolic responses. Individuals who exercise regularly often adopt healthier diets with less processed food, potentially reducing acrylamide exposure. However, post-exercise consumption of energy bars, baked snacks, or coffee-based drinks may increase intake. Thus, the impact of exercise on acrylamide exposure may vary depending on post-exercise nutrition strategies.

### 4.5. Limitations

In this study, body composition was assessed solely using BMI age z-scores, and additional measurements such as waist circumference, waist-to-height ratio (WHtR), and skinfold thickness were not taken. This situation may lead to classification errors in some adolescents, as it may result in an inability to distinguish between fat and muscle mass. However, BMI is the primary anthropometric indicator recommended by the World Health Organization for school-based population screenings and was chosen due to logistical constraints and the need for international comparability. This limitation should be considered when interpreting the study’s findings, and future research should include more comprehensive body composition analyses. The study was limited to only three food groups and, due to its cross-sectional design, no causal relationships were established.

The NEBS scale used is not an assessment tool specifically designed to measure direct acrylamide exposure. The scale measures general dietary behaviors and therefore only indirectly reflects acrylamide intake. In future studies, the use of specific food consumption frequency questionnaires for acrylamide or the evaluation of biomarkers may provide a more accurate measurement of exposure levels.

Since the study has a cross-sectional design, the findings can only be interpreted at the level of association; it is not possible to establish a cause-and-effect relationship. Although significant associations were observed between acrylamide exposure and behavior scores, these findings cannot be evaluated in a causal context.

The study did not collect data on potential confounding factors such as socioeconomic status, parental education level, household income, lifestyle differences, and access to healthy food. These variables could influence both individuals’ dietary and exercise behaviors and their exposure to acrylamide. Therefore, the observed relationships should be interpreted with caution and within a limited context. Including such socioeconomic variables in future studies and controlling for them through multivariate analyses will contribute to more accurate and reliable results.

In this study, a health risk assessment was conducted based solely on the acrylamide levels in bread, simit, tea, coffee, and certain traditional desserts. However, it should be noted that individuals consume different foods together in their daily lives, so the total acrylamide exposure and associated health risks may be higher than the values obtained in this study. Another limitation of this study is the use of a single 24-h dietary recall to estimate acrylamide exposure. This method is prone to recall bias, as participants may not accurately remember their food intake. Such bias may affect the accuracy of exposure estimates. In addition, due to constraints such as time, cost, and the difficulty of obtaining representative food samples, acrylamide analyses were not performed on bread, dessert, and beverage products directly consumed by adolescents. Instead, existing data from recent studies reporting acrylamide levels in these food categories within Türkiye were utilized. Acrylamide levels vary depending on many factors, such as the type and content of the food and the cooking method. Due to time and cost constraints, samples were not taken directly from places where adolescents consume food; instead, a current reference reflecting consumption habits in Türkiye was used. Additionally, consumption amounts vary according to geographical, cultural, and individual differences, which affects both exposure and health risks. This variability explains the differences between the findings of this study and those of other studies in the literature.

## 5. Conclusions

This study revealed that adolescents’ dietary and exercise behaviors are significantly associated with diet-related acrylamide exposure and related health risks. The scores obtained in this study showed that adolescents are in the process of developing healthy eating and exercise habits. Psychological eating tendencies and unhealthy preferences are occasionally observed. Additionally, girls’ NEBS total, HEEB, and MR scores were found to be significantly lower than those of boys. Among the food groups evaluated, bread contributed the most to daily acrylamide intake, especially in boys and individuals with low body weight. Although EDI and THQ values remained within safe limits for most participants, CR values were above acceptable levels, especially in boys and individuals with low body mass index. Low-level but statistically significant positive associations were identified between acrylamide exposure and related health risks and the subscales of the nutrition and exercise behavior scale; particularly, unhealthy and psychological eating behaviors were found to potentially increase acrylamide exposure levels. In conclusion, this study has revealed that adolescents’ dietary and exercise behaviors are not only decisive for their general health status but also for environmental chemical exposure and public health risks. In particular, psychological eating and unhealthy dietary behaviors can increase acrylamide exposure, potentially paving the way for carcinogenic risks. Therefore, comprehensive and multidisciplinary interventions aimed at improving dietary behaviors should be prioritized, especially among the young population. In addition, it is recommended that behavior-based tools such as NEBS be used as part of public health strategies in nutrition education programs for adolescents. NEBS can be an appropriate behavioral assessment tool not only for identifying individuals at risk at the outset, but also for monitoring the impact of intervention programs over time. To enhance the generalizability of the findings and establish causal relationships more robustly, longitudinal studies with larger sample sizes are needed.

## Figures and Tables

**Table 1:** Food groups included in the study and acrylamide levels.

Food Groups	Types of Food	Numbers of Sample	Mean Acrylamide Level	References
Breads	Multi-grain bread	12	79.2 µg/kg	[40,41]
	Whole-meal bread	10	83.5 µg/kg	
	Whole wheat bread	12	76.8 µg/kg	
	Rye bread	8	82.6 µg/kg	
	White bread	10	87.4 µg/kg	
	Rize simit *	6	0.75 µg/1 portion (60 g)	
	Sesame simit *	6	6.35 µg/1 portion (60 g)	
Desserts	Baklava (pistachio)	10	6.49 µg/1 portion (160 g)	[41]
	Künefe	6	5.47 µg/1 portion (175 g)	
	Halka dessert	6	37.0 µg/1 portion (125 g)	
	Lokma dessert	6	11.3 µg/1 portion (100 g)	
	Tulumba dessert	6	12.8 µg/1 portion (100 g)	
Beverages	Black tea	20	3.2 µg/1 cup (80 mL)	[24,42]
	Turkish coffee	8	1.3 µg/1 cup (70 mL)	
	3-in-1 (instant coffee)	12	9.6 µg/1 cup (200 mL)	
	2-in-1 (instant coffee)	8	9.1 µg/1 cup (200 mL)	
	Latte (instant coffee)	8	6.8 µg/1 cup (200 mL)	
	Mocha (instant coffee)	4	14.6 µg/1 cup (200 mL)	
	Americano (ready-to-drink)	8	3.9 µg/1 cup (200 mL)	
	Filter coffee (ready-to-drink)	8	3.3 µg/1 cup (200 mL)	
	Espresso (ready-to-drink)	8	0.8 µg/1 cup (200 mL)	

* Simit is a bakery product mostly consumed for breakfast and is included in the bread group.

**Table 2:** Demographic characteristics of adolescents.

Demographic Characteristics	Number of People (n = 370)	Percentage (%)	Consumption Amounts (Mean ± SD)		
			Bread (g/day)	Desserts (g/day)	Beverage (mL/day)
Gender					
Girls	198	53.5	65 ± 82 ^b^	4 ± 17 ^b^	119 ± 109 ^a^
Boys	172	46.5	134 ± 114 ^a^	11 ± 27 ^a^	123 ± 99 ^a^
BMI					
Underweight	68	18.4	106 ± 113 ^a^	7 ± 24 ^b^	120 ± 104 ^b^
Normal	247	66.8	93 ± 103 ^b^	6 ± 21 ^b^	123 ± 104 ^b^
Overweight/Obese	55	14.8	117 ± 99 ^a^	11 ± 27 ^a^	138 ± 97 ^a^

BMI = Body mass index, SD = Standard Deviation, A z-score of < –2 is classified as underweight, –2 to +1 as normal, +1 to +2 as overweight, and z ≥ +2 as obese [37], ^a, b^ Different letters in the same group indicate statistically significant differences (*p* < 0.05).

**Table 3:** Examination of Statistical Differences Between Demographic Characteristics According to Scale and Subscale Scores.

Demographic Characteristics	NEBS		PAEB		HEEB		UEEB		MR	
	Mean ± SD	Min-Max.	Mean ± SD	Min-Max.	Mean ± SD	Min-Max.	Mean ± SD	Min-Max.	Mean±SD	Min-Max.
Gender										
Girls	129 ± 17.6 ^b^	77–179	29.4 ± 8.75 ^a^	11–53	40.6 ± 8.81 ^b^	18–64	39.0 ± 7.13 ^a^	19–62	20.4 ± 5.04 ^b^	6–30
Boys	137 ± 22.2 ^a^	90–202	29.3 ± 9.98 ^a^	11–55	46.5 ± 9.93 ^a^	18–69	38.7 ± 8.19 ^a^	21–62	22.6 ± 5.15 ^a^	8–30
BMI										
Underweight	132 ± 19.5 ^a^	96–184	30.7 ± 10.2 ^a^	11–54	41.0 ± 8.46 ^a^	26–60	39.2 ± 6.63 ^a^	26–55	21.0 ± 4.95 ^a^	6–30
Normal	133 ± 19.7 ^a^	77–202	28.7 ± 8.81 ^a^	12–51	43.6 ± 9.91 ^a^	18–69	38.6 ± 7.65 ^a^	19–62	21.6 ± 5.24 ^a^	6–30
Overweight/Obese	136 ± 23.6 ^a^	94–198	30.5 ± 10.3 ^a^	16–55	44.8 ± 10.4 ^a^	18–64	39.8 ± 8.72 ^a^	25–62	21.0 ± 5.35 ^a^	8–30

NEBS = Nutrition Exercise Behavior Scale, PAEB = Psychological/dependent eating behavior, HEEB = Healthy nutrition-exercise behavior, UEEB = Unhealthy nutrition-exercise behavior, MR = Meal regularity, BMI = Body mass index, SD = Standard Deviation. A z-score of <–2 is classified as underweight, –2 to +1 as normal, +1 to +2 as overweight, and z ≥ +2 as obese [37], ^a, b^ Different letters in the same group indicate statistically significant differences (*p* < 0.05).

**Table 4:** Comparison of diet-related exposure, THQ, and CR values according to the demographic characteristics of adolescents.

Demographic Characteristics	DE (µg/Day)		BWE (µg/kg/Day)		THQ		CR	
	Mean ± SD	Median (Min–Max)	Mean ± SD	Median (Min–Max)	Mean ± SD	Median (Min–Max)	Mean ± SD	Median (Min–Max)
Bread								
Gender								
Girls	5.65 ± 7.02	2.62 (0–37.2) ^b^	0.11 ± 0.14	0.05 (0–0.70) ^b^	0.05 ± 0.07	0.02 (0–0.35) ^b^	5.52 × 10^−5^ ± 7.00 × 10^−5^ (Potential risk)	2.00 × 10^−5^ (0–3.50 × 10^−4^) ^b^
Boys	13.8 ± 11.9	13.1 (0–50.0) ^a^	0.23 ± 0.21	0.18 (0–0.95) ^a^	0.11 ± 0.11	0.09 (0–0.48) ^a^	1.13 × 10^−4^ ± 1.05 × 10^−4^ (Serious risk)	2.30 × 10^−5^ (0–4.76 × 10^−4^) ^a^
BMI								
Underweight	10.1 ± 10.8	3.50 (0–41.9) ^a^	0.22 ± 0.23	0.08 (0–0.95) ^a^	0.11 ± 0.12	0.04 (0–0.48) ^a^	1.12 × 10^−4^ ± 1.22 × 10^−4^ (Serious risk)	4.07 × 10^−5^ (0–4.76 × 10^−4^) ^a^
Normal	8.84 ± 10.3	3.50 (0–50.0) ^a^	0.15 ± 0.17	0.06 (0–0.82) ^a^	0.08 ± 0.09	0.03 (0–0.41) ^a^	1.13 × 10^−4^ ± 1.06 × 10^−4^ (Serious risk)	8.89 × 10^−5^ (0–4.76 × 10^−4^) ^a^
Overweight/Obese	11.4 ± 10.9	8.24 (0–44.5) ^a^	0.15 ± 0.14	0.10 (0–0.54) ^a^	0.07 ± 0.07	0.05 (0–0.27) ^a^	7.43 × 10^−5^ ± 7.00 × 10^−5^ (Potential risk)	4.90 × 10^−5^ (0–2.68 × 10^−4^) ^a^
Desserts								
Gender								
Girls	0.21 ± 0.77	0 (0–4.06) ^b^	0.00 ± 0.02	0 (0–0.09) ^b^	0 ± 0.01	0 (0–0.04) ^b^	1.94 × 10^−6^ ± 7.44 × 10^−6^ (Potential risk)	0 (0–4.32 × 10^−5^) ^b^
Boys	0.51 ± 1.23	0 (0–7.01) ^a^	0.01 ± 0.02	0 (0–0.09) ^a^	0 ± 0.01	0 (0–0.05) ^a^	4.00 × 10^−6^ ± 9.89 × 10^−6^ (Potential risk)	0 (0–4.51 × 10^−5^) ^a^
BMI								
Underweight	0.34 ± 1.04	0 (0–4.06) ^a^	0.01 ± 0.02	0 (0–0.09) ^a^	0 ± 0.01	0 (0–0.05) ^a^	3.65 × 10^−6^ ± 1.13 × 10^−5^ (Potential risk)	0 (0–4.51 × 10^−5^) ^a^
Normal	0.27 ± 0.87	0 (0–4.06) ^a^	0.00 ± 0.02	0 (0–0.08) ^a^	0 ± 0.01	0 (0–0.04) ^a^	4.04 × 10^−6^ ± 9.94 × 10^−6^ (Potential risk)	0 (0–4.51 × 10^−5^) ^a^
Overweight/Obese	0.67 ± 1.48	0 (0–7.01) ^a^	0.01 ± 0.02	0 (0–0.09) ^a^	0 ± 0.01	0 (0–0.04) ^a^	4.25 × 10^−6^ ± 9.60 × 10^−6^ (Potential risk)	0 (0–4.44 × 10^−5^) ^a^
Beverage								
Gender								
Girls	5.36 ± 5.59	5 (0–23.7) ^a^	0.10 ± 0.11	0.09 (0–0.40) ^a^	0.05 ± 0.05	0.04 (0–0.24) ^a^	5.09 × 10^−5^ ± 5.31 × 10^−5^ (Potential risk)	4.39 × 10^−5^ (0–2.36 × 10^−4^) ^a^
Boys	5.69 ± 5.21	5 (0–22.1) ^a^	0.09 ± 0.09	0.07 (0–0.34) ^a^	0.05 ± 0.04	0.04 (0–0.17) ^a^	4.69 × 10^−5^ ± 4.39 × 10^−5^ (Potential risk)	3.52 × 10^−5^ (0–1.68 × 10^−4^) ^a^
BMI								
Underweight	5.25 ± 4.84	5 (0–18.1) ^a^	0.12 ± 0.11	0.11 (0–0.43) ^a^	0.06 ± 0.06	0.06 (0–0.22) ^a^	6.00 × 10^−5^ ± 5.66 × 10^−5^ (Potential risk)	5.56 × 10^−5^ (0–2.15 × 10^−4^) ^a^
Normal	5.59 ± 5.37	5 (0–23.7) ^a^	0.10 ± 0.09	0.08 (0–0.47) ^ab^	0.05 ± 0.05	0.04 (0–0.24) ^ab^	4.74 × 10^−5^ ± 4.39 × 10^−5^ (Potential risk)	3.57 × 10^−5^ (0–1.68 × 10^−4^) ^ab^
Overweight/Obese	5.49 ± 6.29	5 (0–22.1) ^a^	0.07 ± 0.09	0.06 (0–0.36) ^b^	0.04 ± 0.05	0.03 (0–0.18) ^b^	3.71 × 10^−5^ ± 4.46 × 10^−5^ (Potential risk)	3.17 × 10^−5^ (0–1.82 × 10^−4^) ^b^
Total								
Gender								
Girls	11.2 ± 9.49	9.98 (0–45.8) ^b^	0.21 ± 0.19	0.18 (0–0.86) ^b^	0.11 ± 0.09	0.09 (0–0.43)^b^	1.08 × 10^−4^ ± 9.28 × 10^−5^ (Serious risk)	8.88 × 10^−5^ (0–4.32 × 10^−4^) ^b^
Boys	20.0 ± 14.1	18.1 (0–70.7) ^a^	0.33 ± 0.25	0.29 (0–1.35) ^a^	0.16 ± 0.13	0.14 (0–0.67) ^a^	1.64 × 10^−4^ ± 1.26 × 10^−4^ (Serious risk)	1.43 × 10^−4^ (0–6.74 × 10^−4^) ^a^
BMI								
Underweight	15.7 ± 12.8	13.1 (0–60.7) ^a^	0.35 ± 0.29	0.31 (0–1.35) ^a^	0.18 ± 0.15	0.15 (0–0.67) ^a^	1.76 × 10^−4^ ± 1.47 × 10^−5^ (Serious risk)	1.54 × 10^−4^ (0–6.74 × 10^−4^) ^a^
Normal	14.7 ± 12.2	12.1 (0–61.6) ^a^	0.25 ± 0.21	0.21 (0–0.86) ^b^	0.13 ± 0.10	0.11 (0–0.43) ^b^	1.65 × 10^−4^ ± 1.26 × 10^−4^ (Serious risk)	1.44 × 10^−4^ (0–6.74 × 10^−4^) ^b^
Overweight/Obese	17.6 ± 14.17	15.1 (0–70.7) ^a^	0.23 ± 0.19	0.18 (0–0.85) ^b^	0.12 ± 0.09	0.09 (0–0.43) ^b^	1.16 × 10^−4^ ± 9.39 × 10^−5^ (Serious risk)	8.91 × 10^−5^ (0–4.26 × 10^−4^) ^b^

BMI = Body mass index, SD = Standard Deviation. DE = Daily exposure, BWE = Exposure based on body weight, THQ = Target hazard quotient, CR = Carcinogenic risk, A z-score of <–2 is classified as underweight, –2 to +1 as normal, +1 to +2 as overweight, and z ≥ +2 as obese [37], ^a, b^ Different letters in the same group indicate statistically significant differences (*p* < 0.05).

**Table 5:** Correlation between THQ and CR with acrylamide exposure based on NEBS and subscale scores.

Parameters		Bread				Desserts				Beverage					Total		
		DE (µg/Day)	BWE (µg/kg/Day)	THQ	CR	DE (µg/Day)	BWE (µg/kg/Day)	THQ	CR	DE (µg/Day)	BWE (µg/kg/Day)	THQ	CR	DE (µg/Day)	BWE (µg/kg/Day)	THQ	CR
NEBS	r	0.184	0.168	0.168	0.168	0.118	0.114	0.114	0.114	0.111	0.093	0.093	0.093	0.170	0.152	0.152	0.152
	*p*	0.000 ***	0.001 **	0.001 **	0.001 **	0.023 *	0.029 *	0.029 *	0.029 *	0.033 *	0.075	0.075	0.075	0.001 **	0.003 **	0.003 **	0.003 **
PAEB	r	0.162	0.166	0.166	0.166	0.122	0.120	0.120	0.120	0.020	0.024	0.024	0.024	0.129	0.138	0.138	0.138
	*p*	0.002 **	0.001 **	0.001 **	0.001 **	0.019 *	0.021 *	0.021 *	0.021 *	0.708	0.644	0.644	0.644	0.013 *	0.008 **	0.008 **	0.008 **
HEEB	r	0.100	0.068	0.068	0.068	0.061	0.055	0.055	0.055	0.074	0.033	0.033	0.033	0.095	0.054	0.054	0.054
	*p*	0.055	0.193	0.193	0.193	0.244	0.292	0.292	0.292	0.157	0.521	0.521	0.521	0.067	0.298	0.298	0.298
UEEB	r	0.103	0.103	0.103	0.103	0.169	0.167	0.167	0.167	0.126	0.122	0.122	0.122	0.144	0.143	0.143	0.143
	*p*	0.048 *	0.047 *	0.047 *	0.047 *	0.001 **	0.001 **	0.001 **	0.001 **	0.015 *	0.019 *	0.019 *	0.019 *	0.006 **	0.006 **	0.006 **	0.006 **
MR	r	0.061	0.050	0.050	0.050	−0.064	−0.064	−0.064	−0.064	−0.017	−0.021	−0.021	−0.021	−0.001	−0.010	−0.010	−0.010
	*p*	0.239	0.338	0.338	0.338	0.221	0.221	0.221	0.221	0.743	0.687	0.687	0.687	0.987	0.855	0.855	0.855

* *p* < 0.05, ** *p* < 0.01, *** *p* < 0.01, DE = Daily exposure, BWE = Exposure based on body weight, NEBS = Nutrition Exercise Behavior Scale, PAEB = Psychological/dependent eating behavior, HEEB = Healthy nutrition-exercise behavior, UEEB = Unhealthy nutrition-exercise behavior, MR = Meal regularity.

## Data Availability

The datasets used and/or analysed during the current study are available from the corresponding author on reasonable request.

## Abbreviations

The following abbreviations are used in this manuscript:

WHO	World Health Organization
NEBS	Nutrition Exercise Behavior Scale
PAEB	Psychological/addictive eating behavior
HEEB	Healthy eating-exercise behavior
UEEB	Unhealthy eating-exercise behavior
MR	Meal regularity
CR	Carcinogenic Risk
THQ	Target Hazard Quotient

## References

[1] Canavan C.R., Fawzi W.W. (2019). Addressing Knowledge Gaps in Adolescent Nutrition: Toward Advancing Public Health and Sustainable Development. Curr. Dev. Nutr..

[2] Ayaz-Alkaya S., Kulakçı-Altıntaş H. (2021). Nutrition-Exercise Behaviors, Health Literacy Level, and Related Factors in Adolescents in Turkey. J. Sch. Health.

[3] Micha R., Karageorgou D., Bakogianni I., Trichia E., Whitsel L.P., Story M., Peñalvo J.L., Mozaffarian D. (2018). Effectiveness of school food environment policies on children’s dietary behaviors: A systematic review and meta-analysis. PLoS ONE.

[4] Bull F.C., Al-Ansari S.S., Biddle S., Borodulin K., Buman M.P., Cardon G., Carty C., Chaput J.P., Chastin S., Chou R. (2020). World Health Organization 2020 guidelines on physical activity and sedentary behaviour. Br. J. Sports Med..

[5] World Health Organization (2020). Guidelines on Physical Activity and Sedentary Behaviour.

[6] World Health Organization (2024). Adolescent and Young Adult Health. Erişim Tarihi..

[7] Banfield E.C., Liu Y., Davis J.S., Chang S., Frazier-Wood A.C. (2016). Poor adherence to US dietary guidelines for children and adolescents in the national health and nutrition examination survey population. J. Acad. Nutr. Diet..

[8] Winpenny E.M., Greenslade S., Corder K., van Sluijs E.M.F. (2018). Diet quality through adolescence and early adulthood: Cross-sectional associations of the dietary approaches to stop hypertension diet index and component food groups with age. Nutrients.

[9] Scaglioni S., De Cosmi V., Ciappolino V., Parazzini F., Brambilla P., Agostoni C. (2018). Factors Influencing Children’s Eating Behaviours. Nutrients.

[10] Liu J., Rehm C.D., Onopa J., Mozaffarian D. (2020). Trends in diet quality among youth in the United States, 1999–2016. JAMA.

[11] Roberts C., Steer T., Maplethorpe N., Cox L., Meadows S., Nicholson S., Page P., Swan G. (2018). National Diet and Nutrition Survey: Results from Years 7 and 8 (Combined) of the Rolling Programme (2014/2015–2015/2016).

[12] Han R., Todd A., Wardak S., Partridge S.R., Raeside R. (2023). Feasibility and Acceptability of Chatbots for Nutrition and Physical Activity Health Promotion Among Adolescents: Systematic Scoping Review With Adolescent Consultation. JMIR Hum. Factors.

[13] Dunford E.K., Popkin B.M. (2018). 37 Year snacking trends for US children 1977–2014. Pediatr. Obes..

[14] Liberali R., Kupek E., Assis M.A.A. (2020). Dietary patterns and childhood obesity risk: A systematic review. Child Obes..

[15] Paramasivam A., Murugan R., Jeraud M., Dakkumadugula A., Periyasamy R., Arjunan S. (2024). Additives in Processed Foods as a Potential Source of Endocrine-Disrupting Chemicals: A Review. J. Xenobiot..

[16] Martínez Steele E., Buckley J.P., Monteiro C.A. (2023). Ultra-processed food consumption and exposure to acrylamide in a nationally representative sample of the US population aged 6 years and older. Prev. Med..

[17] Cai C., Song Z., Xu X., Yang X., Wei S., Chen F., Dong X., Zhang X., Zhu Y. (2025). The neurotoxicity of acrylamide in ultra-processed foods: Interventions of polysaccharides through the microbiota-gut-brain axis. Food Funct..

[18] Tareke E., Rydberg P., Karlsson P., Eriksson S., Törnqvist M. (2002). Analysis of acrylamide, a carcinogen formed in heated foodstuffs. J. Agric. Food Chem..

[19] Stadler R.H., Gökmen V. (2024). Acrylamide formation mechanisms. Acrylamide in Food.

[20] Pesce F., Ponzo V., Mazzitelli D., Varetto P., Bo S., Saguy I.S. (2024). Strategies to reduce acrylamide formation during food processing focusing on cereals, children and toddler consumption: A review. Food Rev. Int..

[21] Esposito F., Velotto S., Rea T., Stasi T., Cirillo T. (2020). Occurrence of acrylamide in Italian baked products and dietary exposure assessment. Molecules.

[22] Tajner-Czopek A., Kita A., Rytel E. (2021). Characteristics of french fries and potato chips in aspect of acrylamide content—Methods of reducing the toxic compound content in ready potato snacks. Appl. Sci..

[23] Schouten M.A., Tappi S., Glicerina V., Rocculi P., Angeloni S., Cortese M., Romani S. (2022). Formation of acrylamide in biscuits during baking under different heat transfer conditions. LWT.

[24] Basaran B., Abanoz Y.Y., Boyraz A. (2024). Effects of different brewing conditions on acrylamide levels in Turkish black tea and health risk assessment. J. Food Compos. Anal..

[25] Yashwanth B.S., Premachandran M.S., Karkera P.S., Murthy P.S. (2024). Acrylamide in coffee: Strategies, research and future perspectives. Food Control.

[26] Adimas M.A., Abera B.D., Adimas Z.T., Woldemariam H.W., Delele M.A. (2024). Traditional food processing and Acrylamide formation: A review. Heliyon.

[27] Başaran B., Çuvalcı B., Kaban G. (2023). Dietary acrylamide exposure and cancer risk: A systematic approach to human epidemiological studies. Foods.

[28] EFSA (European Food Safety Authority) (2015). Scientific ppinion on acrylamide in food. EFSA J..

[29] Benford D., Bignami M., Chipman J.K., Ramos Bordajandi L., EFSA (European Food Safety Authority) (2022). Assessment of the genotoxicity of acrylamide. EFSA J..

[30] JECFA (Joint FAO/WHO Expert Committee on Food Additives) (2011). Evaluation of Certain Contaminants in Food: Seventy-Second Report of the Joint FAO/WHO Expert Committee on Food Additives.

[31] European Commission (2019). Commission Recommendation (EU) 2019/1888 of 7 November 2019 on the Monitoring of the Presence of Acrylamide in Certain Foods. https://eur-lex.europa.eu/legal-content/EN/TXT/PDF/?uri=CELEX:32019H1888&from=EN.

[32] Hendekci A., Avcı İ.A. (2020). Adölesanlarda internet bağımlılığı ile beslenme egzersiz davranışları arasındaki ilişki [The relationship between internet addiction and nutrition-exercise behaviors in adolescents]. Ank. Med. J..

[33] Sarı Ç., Ceylan Ç. (2022). Determination of Nutrition Exercise Behaviors of Adolescents and Young Adults in the COVID-19 Pandemic. Mersin Üniv. Tıp Fak. Lokman Hekim Tıp Tarihi Ve Folk. Tıp Derg..

[34] Akdeniz Kudubes A., Ayar D., Bektas İ., Bektas M. (2022). Predicting the effect of healthy lifestyle belief on attitude toward nutrition, exercise, physical activity, and weight-related self-efficacy in Turkish adolescents. Arch. Pediatr..

[35] Biazzi Leal D., Altenburg de Assis M.A., Hinnig P.F., Schmitt J., Soares Lobo A., Bellisle F., Di Pietro P.F., Vieira F.K., de Moura Araujo P.H., de Andrade D.F. (2017). Changes in Dietary Patterns from Childhood to Adolescence and Associated Body Adiposity Status. Nutrients.

[36] World Health Organization (2008). Child Growth Standards: Course Modules and Growth Records—Measuring a Child’s Growth. https://www.who.int/tools/child-growth-standards.

[37] World Health Organization (2025). BMI-For-Age (5–19 years): Interpretation of Cut-Offs. https://www.who.int/tools/growth-reference-data-for-5to19-years/indicators/bmi-for-age.

[38] Yurt S., Save D., Yıldız A. (2016). Adolesanlar için beslenme egzersiz davranışlarını değerlendirme ölçüm aracının geliştirilmesi, geçerliliği ve güvenilirliği. Türkiye Klinikleri. J. Public Health Nu.-Spec. Top..

[39] Taber K.S. (2018). The use of Cronbach’s alpha when developing and reporting research instruments in science education. Res. Sci. Educ..

[40] Basaran B., Anlar P., Oral Z.F.Y., Polat Z., Kaban G. (2022). Risk assessment of acrylamide and 5-hydroxymethyl-2-furfural (5-HMF) exposure from bread consumption: Turkey. J. Food Compos. Anal..

[41] Basaran B., Faiz O. (2022). Determining the levels of acrylamide in some traditional foods unique to Turkey and risk assessment. J Pharm Res..

[42] Başaran B., Aydın F., Kaban G. (2020). The determination of acrylamide content in brewed coffee samples marketed in Turkey. Food Addit. Contam. Part. A.

[43] Basaran B., Sadighara P. (2024). The level, human exposure, and health risk assessment of acrylamide in chips and breakfast cereals: A study from Türkiye. J. Food Compos. Anal..

[44] US EPA (United States Environmental Protection Agency) (1987). Guidance Manual for Assessing Human Health Risks from Chemically Contaminated, Fish and Shellfish [EPA-503/8-89-002].

[45] US EPA (United States Environmental Protection Agency) (2010). Acrylamide. https://cfpub.epa.gov/ncea/iris/iris_documents/documents/subst/0286_summary.pdf#nameddest=rfd.

[46] US EPA (United States Environmental Protection Agency) (2021). Basic Information About the Integrated Risk Information System. https://www.epa.gov/iris/basic-information-about-integrated-risk-information-system.

[47] Mukaka M.M. (2012). Statistics corner: A guide to appropriate use of correlation coefficient in medical research. Malawi Med. J..

[48] Crone E.A., van Duijvenvoorde A.C.K. (2021). Multiple pathways of risk taking in adolescence. Dev. Rev..

[49] Rodríguez-Romo G., Acebes-Sánchez J., García-Merino S., Garrido-Muñoz M., Blanco-García C., Diez-Vega I. (2022). Physical Activity and Mental Health in Undergraduate Students. Int. J. Environ. Res. Public Health.

[50] Schuch F.B., Vancampfort D. (2021). Physical activity, exercise, and mental disorders: It is time to move on. Trends Psychiatry Psychother..

[51] Sampasa-Kanyinga H., Colman I., Goldfield G.S., Janssen I., Wang J., Podinic I., Tremblay M.S., Saunders T.J., Sampson M., Chaput J.P. (2020). Combinations of physical activity, sedentary time, and sleep duration and their associations with depressive symptoms and other mental health problems in children and adolescents: A systematic review. Int. J. Behav. Nutr. Phys. Act..

[52] Güler S., Yavaş Çelik M., Öztürk Çopur E. (2023). Tip 1 diyabetli ergenlerin beslenme ve egzersiz davranışlarının değerlendirilmesi [Evaluation of nutrition and exercise behaviors in adolescents with type 1 diabetes]. J. Educ. Train. Res. (JETR).

[53] Yarar H., Karahan Yılmaz S., Eskici G., Köksal B., Ceylan H.B., Balıkcı R., Saraç O.E. (2023). Adölesan sporcuların beslenme ve egzersiz davranışlarının incelenmesi [Investigation of nutrition and exercise behaviors of adolescent athletes]. CBÜ J. Phys. Educ. Sports Sci..

[54] Aykut T., Avcı P., Kılınçarslan G., Bayrakdar A. (2021). Lise öğrencilerinin beslenme ve egzersiz davranışlarının belirlenmesi [Determination of nutrition and exercise behaviors of high school students]. J. Phys. Educ. Sports Sci..

[55] Erdem E., Efe Y.S., Özbey H. (2023). A predictor of emotional eating in adolescents: Social anxiety. Arch. Psychiatr. Nurs..

[56] Athanasian C.E., Lazarevic B., Kriegel E.R., Milanaik R.L. (2021). Alternative diets among adolescents: Facts or fads?. Curr. Opin. Pediatr..

[57] Dell’Osbel R.S., Donatti T., Henn R.L., Cremonese C., Capp E., Gregoletto M.L.O. (2021). Parental practices, body dissatisfaction and weight control practices in female adolescents from public schools in southern Brazil. Br. J. Nutr..

[58] Hallal P.C., Andersen L.B., Bull F.C., Guthold R., Haskell W., Ekelund U., Lancet Physical Activity Series Working Group (2012). Global physical activity levels: Surveillance progress, pitfalls, and prospects. Lancet.

[59] Keleş B., Göbel P. (2023). Adölesanların Akdeniz Diyetine Uyumu ile Duygusal Yeme ve Stres Durumları Arasındaki İlişkinin Belirlenmesi. Beslenme Ve Diyet Derg..

[60] Mojska H., Gielecińska I., Szponar L., Ołtarzewski M. (2010). Estimation of the dietary acrylamide exposure of the Polish population. Food Chem. Toxicol..

[61] Sirot V., Hommet F., Tard A., Leblanc J.C. (2012). Dietary acrylamide exposure of the French population: Results of the second French Total Diet Study. Food Chem. Toxicol..

[62] Cieslik I., Cieslik E., Topolska K., Surma M. (2020). Dietary acrylamide exposure from traditional food products in Lesser Poland and associated risk assessment. Ann. Agric. Environ. Med..

[63] Zha L., Sobue T., Kitamura T., Kitamura Y., Ishihara J., Kotemori A., Liu R., Ikeda S., Sawada N., Iwasaki M. (2020). Dietary acrylamide intake and the risk of liver cancer: The Japan public health center-based prospective study. Nutrients.

[64] Kito K., Ishihara J., Kotemori A., Zha L., Liu R., Sawada N., Iwasaki M., Sobue T., Tsugane S. (2020). Dietary acrylamide intake and the risk of pancreatic cancer: The Japan public health center-based prospective study. Nutrients.

[65] Basaran B., Abanoz Y.Y., Şenol N.D., Oral Z.F.Y., Öztürk K., Kaban G. (2023). The levels of heavy metal, acrylamide, nitrate, nitrite, N-nitrosamine compounds in brewed black tea and health risk assessment: Türkiye. J. Food Compos. Anal..

[66] Tardiff R.G., Gargas M.L., Kirman C.R., Carson M.L., Sweeney L.M. (2010). Estimation of safe dietary intake levels of acrylamide for humans. Food Chem. Toxicol..

[67] Freisling H., Moskal A., Ferrari P., Nicolas G., Knaze V., Clavel-Chapelon F., Slimani N. (2013). Dietary acrylamide intake of adults in the European Prospective Investigation into Cancer and Nutrition differs greatly according to geographical region. Eur. J. Nutr..

[68] Basaran B. (2024). Estimation of the Dietary Acrylamide Exposure of the Turkish Population: An Emerging Threat for Human Health. Nutrients.

[69] (2019). The Türkiye Nutrition and Health Survey. https://hsgm.saglik.gov.tr/depo/birimler/saglikli-beslenme-ve-hareketli-hayat-db/Dokumanlar/Ingilizce_Yayinlar/TBSA_RAPOR_KITAP_2017_ENG_.pdf.

[70] De Boni A., Pasqualone A., Roma R., Acciani C. (2019). Traditions, health and environment as bread purchase drivers: A choice experiment on high-quality artisanal Italian bread. J. Clean. Prod..

[71] McCullough M.L., Hodge R.A., Um C.Y., Gapstur S.M. (2019). Dietary acrylamide is not associated with renal cell cancer risk in the CPS-II nutrition cohort. Cancer Epidemiol. Biomark. Prev..

[72] Jeong H., Hwang S., Kwon H. (2020). Survey for acrylamide in processed foods from Korean market and individual exposure estimation using a non-parametric probabilistic model. Food Addit. Contam. Part A.

[73] Hirvonen T., Kontto J., Jestoi M., Valsta L., Peltonen K., Pietinen P., Virtamo J. (2010). Dietary acrylamide intake and the risk of cancer among Finnish male smokers. Cancer Causes Control.

[74] Eslamizad S., Kobarfard F., Tsitsimpikou C., Tsatsakis A., Tabib K., Yazdanpanah H. (2019). Health risk assessment of acrylamide in bread in Iran using LC-MS/MS. Food Chem. Toxicol..

[75] Oroian M., Amariei S., Gutt G. (2015). Acrylamide in Romanian food using HPLC-UV and a health risk assessment. Food Addit. Contam. Part B.

[76] Nematollahi A., Kamankesh M., Hosseini H., Ghasemi J., Hosseini-Esfahani F., Mohammadi A., Mousavi Khaneghah A. (2020). Acrylamide content of collected food products from Tehran’s market: A risk assessment study. Environ. Sci. Pollut. Res..

[77] Rakha A., Mehak F., Shabbir M.A., Arslan M., Ranjha M.M.A.N., Ahmed W., Aadil R.M. (2022). Insights into the constellating drivers of satiety impacting dietary patterns and lifestyle. Front. Nutr..

[78] Hyldelund N.B., Frederiksen C., Byrne D.V., Andersen B.V. (2022). Is stress taking the pleasure out of food?—A characterization of the food pleasure profiles, appetite, and eating behaviors of people with chronic stress. Foods.

[79] Lytvynenko O., König L.M. (2024). Investigation of Ukrainian refugees’ eating behavior, food intake, and psychological distress: Study protocol and baseline data. Appl. Psychol. Health Well-Being.

[80] Tomás-Gallego G., Dalmau-Torres J.M., Jiménez-Boraita R., Ortuño-Sierra J., Gargallo-Ibort E. (2025). Adherence to the Mediterranean diet in Spanish university students: Association with lifestyle habits, mental and emotional well-being. Nutrients.

[81] Arsalandeh F., Shemirani F., Nazari M.A., Mirmiran P., Golzarand M. (2025). Effect of low-carbohydrate diets on quality of life, mental health, and speed of memory in adults: A systematic review and meta-analysis of randomised controlled trials. Int. J. Food Sci. Nutr..

[82] Thornley S., Russell B., Kydd R. (2011). Carbohydrate reward and psychosis: An explanation for neuroleptic induced weight gain and path to improved mental health?. Curr. Neuropharmacol..

[83] Lennerz B., Lennerz J.K. (2018). Food addiction, high-glycemic-index carbohydrates, and obesity. Clin. Chem..

[84] Unwin J., Delon C., Giæver H., Kennedy C., Painschab M., Sandin F., Wiss D.A. (2022). Low carbohydrate and psychoeducational programs show promise for the treatment of ultra-processed food addiction. Front. Psychiatry.

[85] Fitrikasari A., Wardani N.D., Sumekar T.A., Saktini F., Asikin H.G., Sulchan M. (2021). The role of psychosocial stressors, carbohydrate and protein intake on serum serotonin and cortisol levels in patients with depression: A preliminary evaluation. Bali Med. J..

[86] Wu Y.K., Pacchioni T.G., Gehi A.K., Fitzgerald K.E., Tailor D.V. (2024). Emotional eating and cardiovascular risk factors in the police force: The Carolina blue project. Int. J. Environ. Res. Public Health.

[87] Clemente-Suárez V.J., Mielgo-Ayuso J., Martín-Rodríguez A., Ramos-Campo D.J., Redondo-Flórez L., Tornero-Aguilera J.F. (2022). The burden of carbohydrates in health and disease. Nutrients.

[88] Basaran A.G., Ozbek Y.D. (2023). A study of the relationship between university students’ food neophobia and their tendencies towards orthorexia nervosa. Behav. Sci..

[89] Jun J., Arendt S.W. (2016). Understanding healthy eating behaviors at casual dining restaurants using the extended theory of planned behavior. Int. J. Hosp. Manag..

[90] Martínez-Vargas L., Vermandere H., Bautista-Arredondo S., Colchero M.A. (2022). The role of social determinants on unhealthy eating habits in an urban area in Mexico: A qualitative study in low-income mothers with a young child at home. Appetite.

[91] Liu Q., Pan F., Luo P., Zhou P. (2025). Levels of acrylamide in food products from a Chinese market and their risk assessment. J. Food Compos. Anal..

[92] Rafacz S.D. (2019). Healthy eating: Approaching the selection, preparation, and consumption of healthy food as choice behavior. Perspect. Behav. Sci..

[93] Kaminsky L.A., German C., Imboden M., Ozemek C., Peterman J.E., Brubaker P.H. (2022). The importance of healthy lifestyle behaviors in the prevention of cardiovascular disease. Prog. Cardiovasc. Dis..

[94] Diab A., Dastmalchi L.N., Gulati M., Michos E.D. (2023). A heart-healthy diet for cardiovascular disease prevention: Where are we now?. Vasc. Health Risk Manag..

[95] Lee Y., Kim T., Jung H. (2022). The relationships between food literacy, health promotion literacy and healthy eating habits among young adults in South Korea. Foods.

[96] Ljubičić M., Sarić M.M., Rumbak I., Barić I.C., Sarić A., Komes D., Guiné R.P. (2022). Is better knowledge about health benefits of dietary fiber related to food labels reading habits? A Croatian overview. Foods.

[97] Leech R.M., Worsley A., Timperio A., McNaughton S.A. (2015). Understanding meal patterns: Definitions, methodology and impact on nutrient intake and diet quality. Nutr. Res. Rev..

[98] Aljuraiban G.S., Chan Q., Oude Griep L.M., Brown I.J., Daviglus M.L., Stamler J., INTERMAP Research Group (2015). The impact of eating frequency and time of intake on nutrient quality and body mass index: The INTERMAP study, a population-based study. J. Acad. Nutr. Diet..

[99] Al-Sowayan N.S., Almeneay B., Al Othaim T. (2023). Effect of Low and High Glycemic Index Meals on Hunger and Satiety. Adv. Biosci. Biotechnol..

[100] Oliveira G.A.L., Santos Gonçalves V.S., Nakano E.Y., Toral N. (2024). Consumption of ultra-processed foods and low dietary diversity are associated with sedentary and unhealthy eating behaviors: A nationwide study with Brazilian Schoolchildren. PLoS ONE.

[101] Kang Y., Kang M., Lim H. (2024). Age-specific association between meal-skipping patterns and the risk of hyperglycemia in Korean adults: A national cross-sectional study using the KNHANES data. BMC Public Health.

[102] Galdino-Silva M.B., Almeida K.M.M., Oliveira A.D.S.D., Santos J.V.L.D., Macena M.D.L., Silva D.R., Bueno N.B. (2024). A Meal with Ultra-Processed Foods Leads to a Faster Rate of Intake and to a Lesser Decrease in the Capacity to Eat When Compared to a Similar, Matched Meal Without Ultra-Processed Foods. Nutrients.

[103] Ducrot P., Méjean C., Aroumougame V., Ibanez G., Allès B., Kesse-Guyot E., Péneau S. (2017). Meal planning is associated with food variety, diet quality and body weight status in a large sample of French adults. Int. J. Behav. Nutr. Phys. Act..

[104] Leech R.M., Livingstone K.M., Worsley A., Timperio A., McNaughton S.A. (2016). Meal frequency but not snack frequency is associated with micronutrient intakes and overall diet quality in Australian men and women. J. Nutr..

[105] Almoraie N.M., Alothmani N.M., Alomari W.D., Al-Amoudi A.H. (2025). Addressing nutritional issues and eating behaviours among university students: A narrative review. Nutr. Res. Rev..

